# Identification of novel inhibitors of Keap1/Nrf2 by a promising method combining protein–protein interaction-oriented library and machine learning

**DOI:** 10.1038/s41598-021-86616-1

**Published:** 2021-04-01

**Authors:** Yugo Shimizu, Tomoki Yonezawa, Junichi Sakamoto, Toshio Furuya, Masanori Osawa, Kazuyoshi Ikeda

**Affiliations:** 1 Division of Physics for Life Functions, Faculty of Pharmacy, Keio University, 1-5-30 Shibakoen, Minato-ku, Tokyo 105-8512 Japan; 2 Lifematics Inc., Sanshin-Hatchobori Bldg. 5F, 2-25-10 Chuo-ku, Hatchobori, Tokyo 104-0032 Japan; 3 Axcelead Drug Discovery Partners, Inc., 26-1, Muraoka-Higashi 2-chome, Fujisawa, Kanagawa 251-0012 Japan; 4 Drug Discovery Department, Research and Development Division, PharmaDesign, Inc., Hatchobori 2-19-8, Chuo-ku, Tokyo 104-0032 Japan

**Keywords:** Drug discovery and development, Machine learning, Computational chemistry, Screening, Chemical libraries

## Abstract

Protein–protein interactions (PPIs) are prospective but challenging targets for drug discovery, because screening using traditional small-molecule libraries often fails to identify hits. Recently, we developed a PPI-oriented library comprising 12,593 small-to-medium-sized newly synthesized molecules. This study validates a promising combined method using PPI-oriented library and ligand-based virtual screening (LBVS) to discover novel PPI inhibitory compounds for Kelch-like ECH-associated protein 1 (Keap1) and nuclear factor erythroid 2-related factor 2 (Nrf2). We performed LBVS with two random forest models against our PPI library and the following time-resolved fluorescence resonance energy transfer (TR-FRET) assays of 620 compounds identified 15 specific hit compounds. The high hit rates for the entire PPI library (estimated 0.56–1.3%) and the LBVS (maximum 5.4%) compared to a conventional screening library showed the utility of the library and the efficiency of LBVS. All the hit compounds possessed novel structures with Tanimoto similarity ≤ 0.26 to known Keap1/Nrf2 inhibitors and aqueous solubility (AlogP < 5). Reasonable binding modes were predicted using 3D alignment of five hit compounds and a Keap1/Nrf2 peptide crystal structure. Our results represent a new, efficient method combining the PPI library and LBVS to identify novel PPI inhibitory ligands with expanded chemical space.

## Introduction

Protein–protein interactions (PPIs) have received increasing attention as attractive targets for drug discovery over the past decade^1^. However, unlike major drug targets such as kinases and G protein-coupled receptors (GPCRs), PPIs are recognized as intractable targets^2^ because the properties required for PPI-modulating molecules are different from those for small-molecule drugs that follow Lipinski's rule of five (*i.e.*, molecular weight < 500, etc*.*)^3^. PPI interfaces are relatively larger than the average interaction areas of single protein targets, and thus molecules inhibiting PPIs tend to be larger and have diverse conformations^4^. High-throughput screening (HTS) is commonly used to find active compounds in early drug discovery processes, but it has been shown that the rate of obtaining hit compounds is significantly low for HTS targeting PPIs using a chemical library composed of small-molecule compounds^5^. Therefore, there is a need for a focused library specific to PPIs, for example, consisting of small-to-medium-sized molecules with properties that extend the rule of five (*e.g.*, molecular weight ranging from 450 to 750)^6^, and compounds that mimic secondary structures (α-helix and β-sheet) and 3D structures of function-related motif sequences on the interfaces. Recently, a PPI-oriented library, called DLiP, was developed in the Japan Agency for Medical Research and Development (AMED) project^7^. The DLiP library was designed based on the 3D structure of the interface of 117 diverse PPI complexes and the physicochemical properties of known PPI inhibitors. It consists of small-to-medium-sized (molecular weight ranging from 450 to 650) compounds with new synthetic structures selected from a virtual library of over 6 million commercially available (synthesizable) compounds. However, the small-to-medium-sized molecules have not sufficiently been validated so far by assays against specific PPI targets.

Experimental validation of all compounds in such a PPI library is not practical because of the high cost. To find hit compounds efficiently from a library, in silico methods have been widely used in drug development^8^. Virtual screening (VS) is a standard procedure in the discovery of hit and lead compounds that enables computational evaluation of the activity of a large number compounds with unknown activity^9^. VS is mainly classified into two methods: structure- and ligand-based VS (SBVS and LBVS, respectively)^10,11^. SBVS involves docking of compounds into the target protein structure, whereas LBVS uses activity information of known ligands to create prediction models without requiring the knowledge of protein crystal structures. Such an in silico approach is also important in the rational design of PPI-modulating molecules. Recently, the discovery of novel inhibitors of some PPI targets using a ligand-based approach was successful^12,13^. For example, Melagraki et al.^12^ combined SBVS and LBVS to discover novel small-molecule PPI inhibitors of tumor necrosis factor (TNF) and receptor activator of nuclear factor κB ligand (RANKL). They created a ligand-based model from 2,481 known TNF inhibitors using majority vote of outputs from three machine learning algorithms: k-nearest neighbor, nearest neighbor, and random forest (RF), and used it to analyze compounds shortlisted using SBVS of 14,400 commercial compounds. Reker et al.^13^ created RF models of CXC chemokine receptor 4 and its endogenous ligand CXCL-12 from 287 curated ligands, which were used to analyze 1,465,960 compounds from an HTS compound collection to filter compounds used for bioassay. Furthermore, they included the results of their bioassay into the known activity dataset to refine the model and further obtain hits. We conceived that a similar approach using LBVS could efficiently streamline the compounds in a PPI library to a limited number, to use in an experimental validation of the library for a specific PPI target, which leads to discovery of hit compounds at a lower cost.

To validate the utility of a PPI library for practical screenings and the effectiveness of ligand-based predicting methods for a PPI library, we focused on Kelch-like ECH-associated protein 1 (Keap1) and nuclear factor erythroid 2-related factor 2 (Nrf2) as a desirable PPI target. Nrf2 is a major transcription factor that protects cells from oxidative stress^14^ and inflammatory responses^15^. Therefore, Nrf2 is involved in various diseases such as cancer^16^, neurodegenerative diseases (*e.g.*, Alzheimer's Disease^17,18^, Parkinson’s disease^17,19^, and Huntington’s disease^20^), diabetes^21^, liver disease^22^, respiratory disease^23^, sepsis^24^, and other diseases^25,26^. The Kelch domain of Keap1 binds to the Neh2 domain of Nrf2, which leads to ubiquitination and subsequent degradation of Nrf2^27^. Thus, Keap1 acts as a negative regulator of Nrf2. Inhibition of their PPI activates Nrf2 and is a promising therapeutic target for diseases such as neurodegenerative disease, diabetes, liver disease, and sepsis.

Although many electrophilic molecules that target the cysteine residues in Keap1 to activate Nrf2 have been found, most cause off-target problems due to an indiscriminate binding to cysteine residues of other proteins^28,29^. Hence, direct inhibitors of the Keap1/Nrf2 PPI are desired. A number of Keap1/Nrf2 PPI inhibitors have been discovered recently by screening using several strategies^30^ such as HTS^31,32^, SBVS^33–36^, and fragment-based approaches^37^. However, no drugs for direct Keap1/Nrf2 PPI inhibition have been approved to date^38^; therefore, discovering novel inhibitors with new scaffolds and increasing chemical diversity using a compound library in screening would be useful in drug development. The DLiP library contains new and diverse compounds designed for PPI targets, and thus it is a good candidate as a screening library for Keap1/Nrf2 PPI. Meanwhile, use of LBVS in the selection of screening compounds is expected to uncover a variety of hit compounds other than SBVS. LBVS for Keap1/Nrf2 PPI needs a number of known ligands validated in experimental assays. In this study, we obtained activity data of Keap1/Nrf2 PPI ligands from public databases such as ChEMBL^39^ and TIMBAL^40^, and collected a potentially suitable number of known active/inactive compounds for LBVS using machine learning techniques. Hence, combining the newly constructed PPI library, DLiP, and LBVS may be prospectively used to discover new PPI inhibitors of Keap1/Nrf2; its effectiveness is demonstrated in this paper.

## Results and discussion

### Overview of discovering new Keap1/Nrf2 inhibitors using a PPI-oriented library combined with LBVS

**Figure 1:**
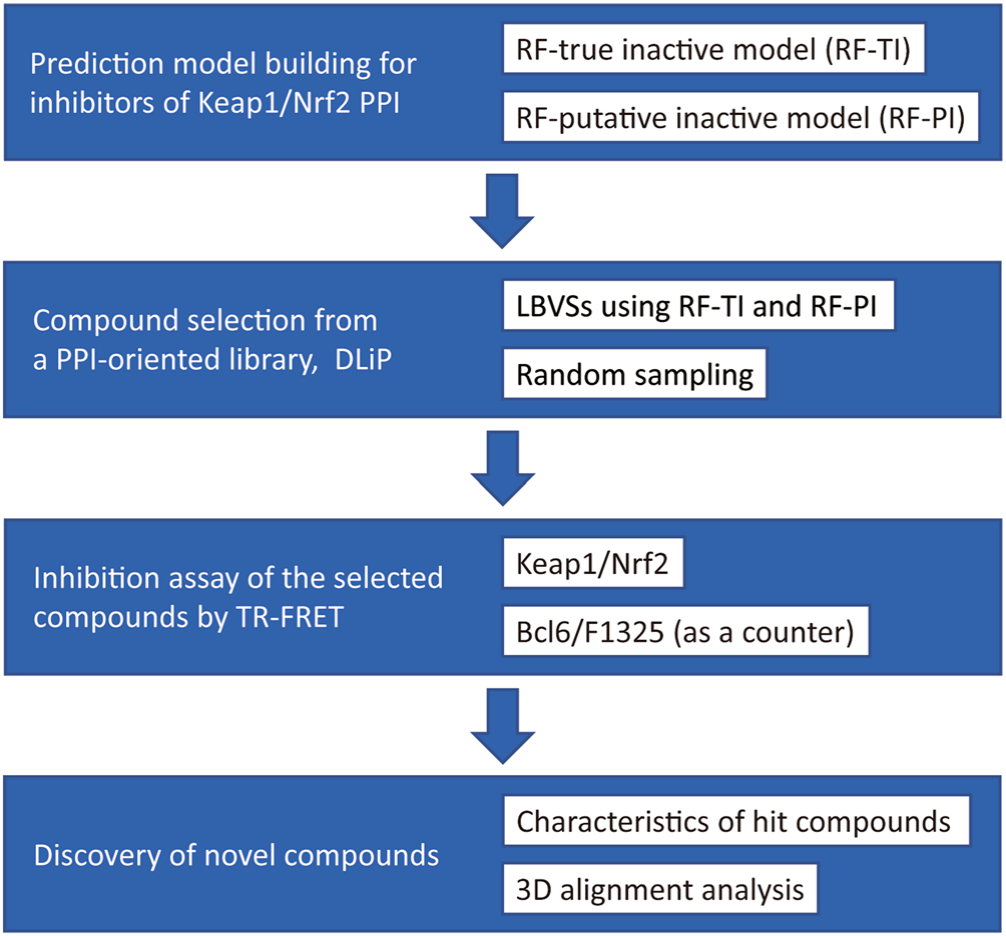
Overview of the process to discover new Keap1/Nrf2 inhibitors.

First, we created two RF models distinguishing known Keap1/Nrf2 inhibitors from non-inhibitors or general screening compounds (Fig. 1). Then, prospective Keap1/Nrf2 inhibitor compounds were selected using LBVS with the two RF models from a PPI-oriented library. To evaluate the effectiveness of LBVS and the utility of the PPI library, additional compounds were randomly sampled from the library. Finally, the inhibitory activities of the selected compounds were validated using time-resolved fluorescence resonance energy transfer (TR-FRET) assays for Keap1/Nrf2 and counter TR-FRET assays for B-cell lymphoma 6 (Bcl6)/F1325.

### Two RF models using different types of negative data

With an aim to build models predicting Keap1/Nrf2 PPI inhibitory activity of compounds, we used an RF classification algorithm. RF is a supervised machine learning method that uses numerous decision trees^41^. RF is a common, easy-to-use method because it requires few parameter adjustments and has a fast computation run time using parallel processing. To expand the scope of the learning, we created two RF models: RF-true inactive (RF-TI) and RF-putative inactive (RF-PI). While the RF-TI model was generated from active and inactive compounds for Keap1/Nrf2 comprehensively collected from three public databases, the RF-PI model was from active compounds in the ChEMBL database and putative inactive compounds. The use of putative inactive compounds as negative training data is known as an alternative strategy for dataset preparation in LBVS^42,43^. Most compounds in large compound libraries are not tested for particular targets and are generally assumed to have a low likelihood of being active and are used as putative inactive compounds. In this study, we used commercial compounds as the putative inactives in the RF-PI model. Although training dataset using true inactives tends to be imbalanced (*i.e.*, the numbers of actives and inactives are not equal) for a target in general, that of RF-TI was inadvertently almost balanced. In contrast, use of putative inactives enables the training dataset to be balanced. Therefore, the result from the RF-PI model could be used as a rough guide in the expansion of models for other general targets (*e.g.*, > 1,500 targets in ChEMBL). To evaluate the performance of the RF models, the average performance scores of seven basic metrics were calculated using fivefold cross-validation that randomly split their original training dataset to generate five pairs of training and test sets (Table 1, please refer to the Materials and Methods Section for the details). All the metrics showed high values, indicating good performance of the two RF models against similar compounds used in the training. The high performance of the RF-PI model is reasonable because the difference between actives and putative inactives tend to be large, and the classifying task would be relatively easy.

**Table 1:** The average performance scores of the RF models in fivefold cross-validation, where the original training set of each RF model was split into five pairs of training and test sets (please refer to the Materials and Methods Section for the details).

Model	Accuracy	Precision	Recall	Specificity	MCC	AUCROC	AUCPR
RF-TI	0.79	0.81	0.76	0.81	0.57	0.85	0.86
RF-PI	0.99	0.98	1.00	0.98	0.98	1.00	1.00

*MCC* Matthews correlation coefficient, *AUCROC* the area under the receiver operating characteristic curve, *AUCPR* the area under the precision-recall curve.

### Compound selection for Keap1/Nrf2 assay using two RF models and random sampling

**Figure 2:**
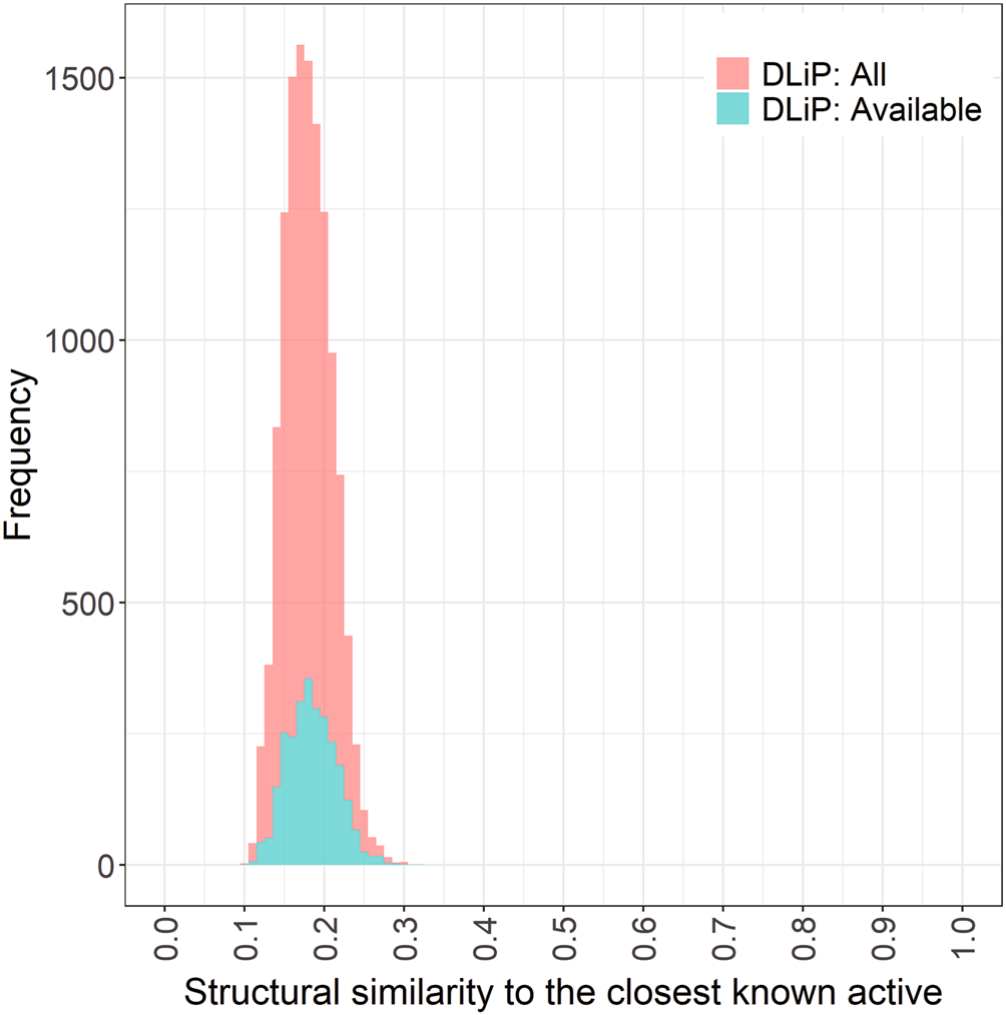
Distribution of structural similarities (Tanimoto similarities of FCFP_6) of all (N = 12,593, light red) compounds of the DLiP library and their available subset in this study (N = 2,684, cyan) to their closest known Keap1/Nrf2 PPI inhibitors in the databases.

**Figure 3:**
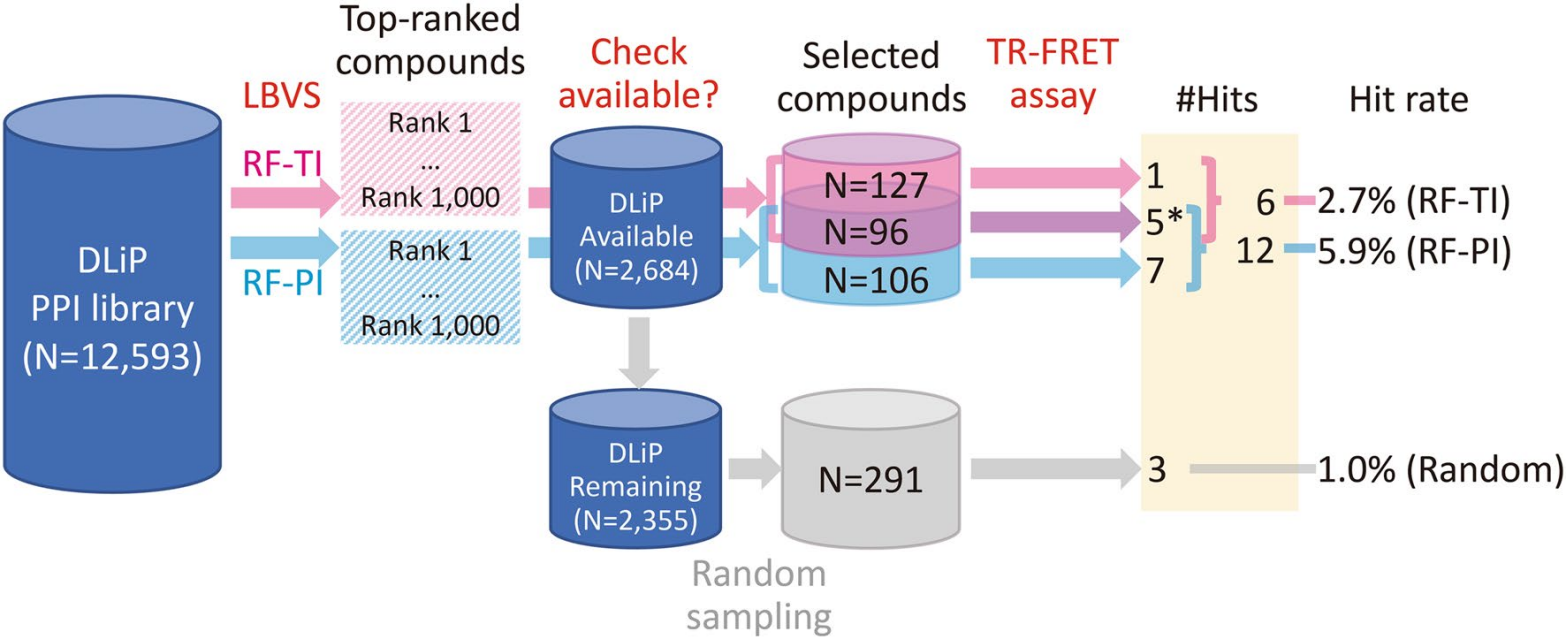
A diagram of the flow from compound selections to TR-FRET assay results. The number of hit compounds marked by asterisk includes a non-specific hit.

We used a PPI-oriented library, the DLiP library, as a compound source to search Keap1/Nrf2 PPI inhibitors. All compounds in the DLiP library have low similarity (< 0.32) to the known Keap1/Nrf2 inhibitors (Fig. 2), and thus should serve as a good source in discovering Keap1/Nrf2 PPI inhibitors with novel structures. We selected candidate compounds from the DLiP library using the RF models and random sampling, and tested their Keap1/Nrf2 PPI inhibitory activities by bioassay (Fig. 3). To select the candidate compounds, probability scores of potential Keap1/Nrf2 PPI inhibitors were calculated for a total of 12,593 compounds in the DLiP library using the two RF models, and the compounds were sorted in descending order based on the prediction scores. The top 1,000 compounds ranked using each model were selected as bioassay candidates. In practice, 2,684 compounds from the DLiP library had been synthesized, and were available for bioassay in October 2019. The diversity of the structural similarities of the available subset to the known Keap1/Nrf2 inhibitors did not decrease compared to that of all the DLiP library sets (Fig. 2). After being filtered based on availability, 329 of the 2,684 compounds were selected for bioassay, consisting of 96 that overlapped in both models, and 106 and 127 in the RF-PI and RF-TI models, respectively also indicating that different scopes between the two RF models are expected (Supplementary Fig. S1). Furthermore, 20.2% and 22.3% of available compounds were from the high-ranked selection by RF-PI and RF-TI, respectively, which was not different from the percentage (2,684 of 12,593, 21.3%) of availability of the whole set. Therefore, the 329 selected compounds were not biased in terms of the prediction results. In addition to these compounds, 291 other compounds were randomly sampled from the remaining 2,355 compounds for comparison. In total, 620 compounds were selected for the bioassay.

### Keap1/Nrf2 TR-FRET assay

The 620 compounds were tested using TR-FRET to evaluate their inhibitory activities against the Keap1/Nrf2 PPI. The TR-FRET assay resulted in 16 hit compounds with the inhibition rate > 15% at 100 μM concentration (Table 2), among which compounds **1** and **2** exhibited a high inhibition rate (85.5% and 74.3%, respectively). A counter assay using Bcl6/F1325 was also performed to confirm the specificity of the tested compounds (Table 2). Only compound **1** exhibited a high inhibition rate (65.2%) at 100 μM in the counter assay, which suggests that it is a non-specific inhibitor. Finally, 15 compounds were identified as Keap1/Nrf2 PPI inhibitors.

**Table 2:** Hit compounds in TR-FRET assays for Keap1/Nrf2.

	Structure	TR-FRET (Keap1/Nrf2)^*a*^ (%)	TR-FRET (Bcl6/ F1325)^*a*^ (%)	Rank (RF-TI)^*b*^	Rank (RF-PI)^*b*^	Structural similarity^*c*^
1		85.5	65.2	129^*^	44^*^	0.228
2		74.3	NI	1320	419^*^	0.253
3		47.0	4.9	2604	426^*^	0.241
4		29.9	NI	905^*^	775^*^	0.262
5		29.2	NI	19^*^	70^*^	0.256
6		27.1	NI	6830	326^*^	0.155
7		22.1	NI	1253	242^*^	0.175
8		22.1	5.1	44^*^	51^*^	0.258
9		18.5	NI	1578	3366	0.141
10		18.3	NI	1475	256^*^	0.164
11		18.2	1.0	1868	525^*^	0.233
12		18.1	6.4	1365	3382	0.143
13		17.7	NI	10,755	527^*^	0.151
14		17.2	NI	497^*^	274^*^	0.170
15		16.8	NI	898^*^	1946	0.234
16		15.2	NI	3,197	5548	0.138

^*a*^Inhibition rates (%) of the compounds at 100 μM concentration of TR-FRET assays for Keap1/Nrf2 and Bcl6/F1325 (counter assay) are shown as mean value of two measurements. *NI* no inhibition.

^*b*^The prediction results using two RF models (RF-TI and RF-PI) are shown as the rank of the prediction scores. The ranks that satisfied the selecting threshold (*i.e.*, ≤ 1000) are marked with asterisks.

^*c*^Structural similarities to the closest known Keap1/Nrf2 PPI inhibitors are shown.

### Characteristics of hit compounds

**Figure 4:**
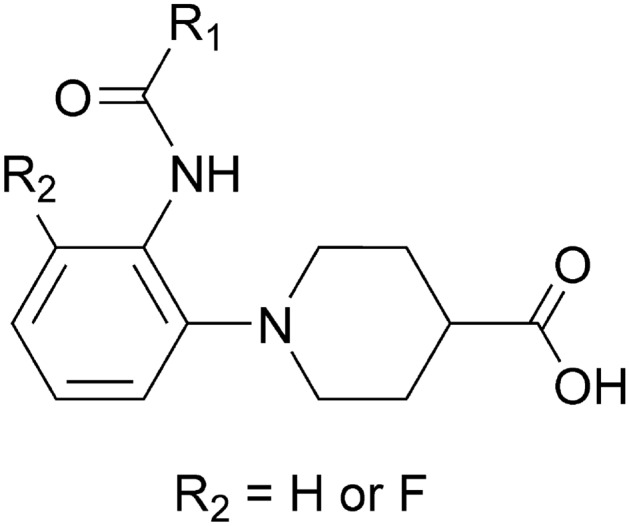
A common substructure of five hit compounds (**2**, **3**, **5**, **8**, and **11**). The uncommon part is shown as R_1_.

The 15 hit compounds were separated into two groups based on their molecular weights (450–500 and 610–650). AlogP of most hit compounds (13 out of 15) were < 5, suggesting good aqueous solubility. The combinations of molecular weight and AlogP of the hit compounds were also widely distributed, showing their diversity in the molecular properties (Supplementary Fig. S2). The structures of the hit compounds are distinct from known Keap1/Nrf2 inhibitors. Specifically, the structural similarities between the hit compounds and their closest compounds in the known Keap1/Nrf2 inhibitors were 0.14–0.26 (Table 2). Compounds **2**, **3**, **5**, **8**, and **11** have the same substructure of an *ortho*-substituted aromatic amide with a carboxylated piperidine (Fig. 4). Of the assayed 620 compounds, 17 have this substructure, and 29.4% (5/17) of tested compounds having the substructure were hit. The common substructure does not exist in the known Keap1/Nrf2 inhibitors and rarely (< 0.001%) exist in the ChEMBL database, indicating the novelty of the five hit compounds in terms of substructure.

### Effectiveness of in silico predictions

Among the 16 hit compounds, including one non-specific inhibitor, twelve, six, and three were selected by RF-PI, RF-TI, and random sampling, respectively (Table 2, Fig. 3). The hit rates of the RF-PI and RF-TI models, and random sampling were 5.9%, 2.7%, and 1.0%, respectively. Assuming that the hit rate of 2,064 compounds not used for the assay was same as the random hit rate (1.0%), the number of total possible hits would be 16 + 2064 × 1.0/100 = 37. The possible hit rate of a random sampling from all the available compounds in the library was estimated as 37/2684 = 1.4% (36/2684 = 1.3% for specific hits), which indicates that the two RF models were effective. The structural similarities (Tanimoto similarities of FCFP_6)^44^ of the 13 hits obtained by RF models to their closest known inhibitors were 0.15–0.26 (Table 2) and their distribution was not biased in the highest similarity region (~ 0.32, Fig. 2), which indicates difficulty in finding them by structural similarity alone, and the power of the machine learning method. On the other hand, the other three hits were not high-ranked by the RF models and obtained by random sampling. This may be because of innate difficulty in prediction using machine learning for those having relatively low structural similarities (≤ 0.143) to known inhibitors. Surprisingly, the RF-PI model resulted a higher hit rate than the RF-TI model, despite the use of a relatively small number of actives and no true inactives in the learning. This seems to be caused by the limited applicability of the model due to the low structural similarities (< 0.32 and < 0.35, respectively, Fig. 2 and Supplementary Fig. S3) between all the DLiP library compounds to the known Keap1/Nrf2 PPI databases’ actives and inactives, and the high structural similarities (~ 0.62 on average) between the known actives and their closest known inactives. Considering that many drug targets have insufficient inactives reported, the use of putative inactives in model building would be a better option in LBVS for other targets than Keap1/Nrf2.

### Utility of the PPI library

Marcotte et al.^35^ performed HTS and counter screening for Keap1/Nrf2 PPI against 267,551 compounds of the Evotec Lead Discovery library and 1,911 compounds selected by SBVS from three vendor catalogs. Their screening resulted in 18 hit compounds and a hit rate of 0.0067% for a threshold of 21% inhibition at a concentration of 50 μM. The specific hit rate of the DLiP library was at least 0.12% (15/12,593). Because the available subset seemed not to be biased compared to the whole library in terms of the prediction result and structural similarity to the known inhibitors, the hit rate of the DLiP library was estimated to be at least 0.56% (15/2,684). Moreover, the possible hit rate of the DLiP library was calculated as 1.3% if the hit rate of the remaining available compounds (N = 2,064) was same as the random sampling (1.0%). Therefore, the hit rate of the DLiP library was estimated to be 0.56–1.3%, suggesting that the DLiP library had a high hit rate for Keap1/Nrf2 PPI in comparison with that using a general library as mentioned above even though the difference of the thresholds for hit identification was considered.

### Ligand–protein Interaction predicted by 3D alignment

**Figure 5:**
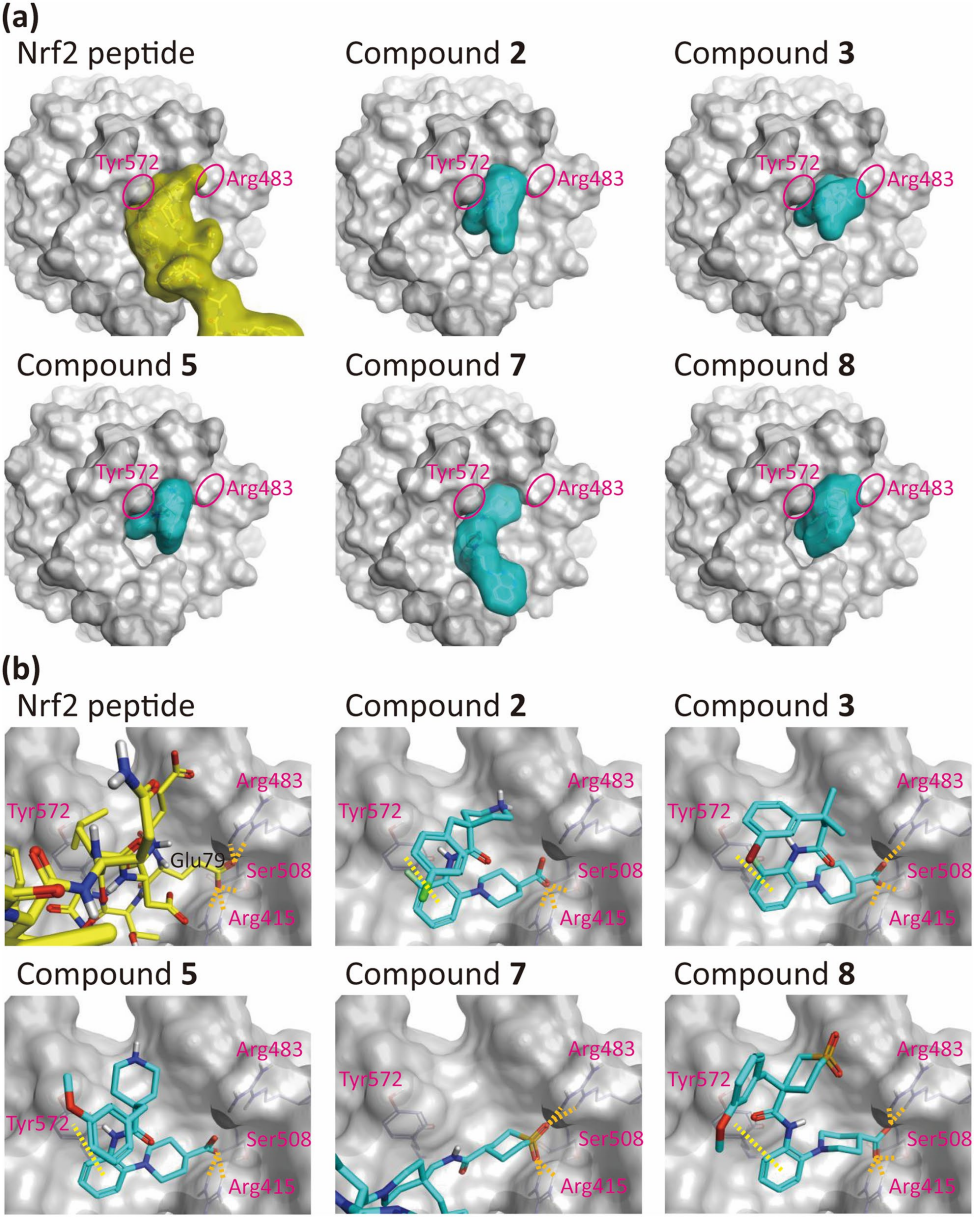
A binding pose of Nrf2 peptide to Keap1 protein (PDB ID: 2flu)^45^ and predicted binding poses of five hit compounds (**2**, **3**, **5**, **7**, and **8**) to Keap1 based on 3D alignment. (**a**) The compounds fitted the binding pocket of Keap1. (**b**) Acceptor interaction (orange dashed lines) between carboxy terminus of the five compounds and Keap1 (Arg415, Arg483, and Ser508), and π-stacking interaction (yellow dashed lines) between compound (**2**, **3**, **5**, and **8**) and Keap1 (Tyr572) were observed.

To deepen the understanding of the molecular structure–activity relationship of the hit compounds, we selected five hit compounds (**2**, **3**, **5**, **7**, and **8**) with inhibition rates > 20% at 100 μM for Keap1 and no inhibition for the counter assay, and constructed their 3D alignment models against an X-ray crystal structure of Keap1/Nrf2 peptide (PDB ID: 2flu)^45^. The 3D alignment models showed the fit of the five hit compounds into the binding pocket of Keap1 (Fig. 5a). It has been shown that hydrogen bond acceptors (Glu79 and Glu82) of Nrf2 interact with Keap1 at Arg415, Arg483, and Ser508; Ser363, Arg380, and Asn382, respectively^45^. Moreover, these interactions contribute to high binding affinity between Keap1 and the known ligands^46^. The binding poses derived from 3D alignment models showed similarities to the X-ray structures in the former acceptor interaction to Keap1 (Fig. 5b). Tyr572 of Keap1 forms a π-stacking interaction with several known ligands^5,35^. The interaction was also observed in the binding poses of hit compounds (**2**, **3**, **5**, and **8**) and the contributing aromatic ring was in their common substructure (Figs. 4 and 5b). These observations suggested that our hit compounds are reasonable as Keap1/Nrf2 inhibitors. However, quantitative activity analysis using highly potent compounds would be required in further verification studies.

## Conclusions

We performed LBVS against a PPI library, DLiP, for Keap1/Nrf2 PPI, which identified 12 specific hits in the TR-FRET assay. In addition, random sampling of the DLiP library identified three other hits. The comparison of these results showed that our prediction method using two RF models was an effective screening method. Moreover, the DLiP library had a higher rate of identifying Keap1/Nrf2 PPI inhibitors than the general (*i.e.*, not focused on PPI) large library for HTS. Considering the diversity of the interfaces of referenced targets of the DLiP library, it would also be useful for general PPI targets.

The DLiP library is a collection of newly synthesized compounds with numerous diverse chemical structures including novel substructures, and thus an attractive source for finding hit compounds. All the compounds in the DLiP library had structures that were dissimilar to those of previously known Keap1/Nrf2 PPI inhibitors. In general, it is difficult to predict the activity of compounds when their chemical space is distant from the training set’s chemical space; nevertheless our predicting methods could identify compounds with novel structures including a unique substructure, showing their practical applicability. In addition, we demonstrated the effectiveness of the predicting method using a relatively small number of actives and putative inactives, suggesting that the method would be applicable for a wide range of targets.

The inhibition rate of the hit compounds not being high in this study remains a concern. The increase of known activity data usually enables creating new and more efficient prediction models. More hits can be identified by creating refined models using our assay results, which helps finding active compounds with high activity. Moreover, increase in hits (including similar substructures) leads to the creation of effective regression models that directly predict the quantitative activity of compounds, which is further beneficial. Taken together, the findings of this study present a new efficient method for investigating novel ligands with PPI inhibitory activity.

## Materials and methods

### PPI library

We recently developed a chemical library, the DLiP library, consisting of 12,593 small-to-medium-sized molecules that target PPI inhibition. The DLiP library was designed based on the 3D structure of the PPI interface and the physicochemical properties of various known PPI inhibitors. It should be noted that the reference PPI targets used for the library construction did not include Keap1/Nrf2, and thus the library could be used as a general PPI library for Keap1/Nrf2 PPI. The molecular weights of the designed compounds mainly ranged from 450 to 650. To ensure the quality and diversity of the library, compounds with spherical or new scaffold structures were selected based on the principal moment of inertia. All the compounds passed pan-assay interference compounds (PAINS) filters^47^ that are an assembly of unfavorable substructures for screening.

### Data collection for machine learning

The compounds experimentally tested for Keap1/Nrf2 PPI inhibition and their activity values were found and obtained from three public databases: ChEMBL, TIMBAL, and 2P2I^48^. We defined the binary activity classification, active or inactive, for those compounds based on the activity values. Specifically, compounds from ChEMBL and TIMBAL with the minimum standardized activity values corresponding to ≤ 10 μM were defined as active samples, and those with higher values were defined as inactive samples. In contrast, only active samples were extracted from the 2P2I database.

A putative inactive dataset was created by randomly selecting vendor (Maybridge) compounds prepared in Pipeline Pilot (Dassault Systèmes BIOVIA, BIOVIA Pipeline Pilot, release 2018, San Diego, Dassault Systèmes, 2018). To avoid misplacement of true actives in the putative inactive dataset, the compounds with structures that were identical to any active compounds were excluded from the vendor catalog.

### Calculation of molecular descriptors and structural similarity

The chemical structures of the curated compounds were obtained in the SDF format. The structures of the largest fragment were used in the following calculation of compound descriptors. Five molecular properties (molecular weight; AlogP; and number of H acceptors, H donors, and rotatable bonds) and functional structural fingerprints (FCFP_6^49^) of the compounds were calculated using Pipeline Pilot. Structural similarity between compounds was calculated by Tanimoto similarity (*i.e.*, Tanimoto coefficient) of the FCFP_6 bits.

### Machine learning

RF models classifying active and inactive compounds for Keap1/Nrf2 PPI were created using molecular properties and fingerprints as features. The calculation was performed using the R package ranger 0.10.1^50,51^ that enables fast computation against datasets with numerous features. We used 1,000 trees in the models and the default number of features was set for node splitting (square root of the number of features). We created two RF models from different datasets. The first (RF-PI) was created using 52 Keap1/Nrf2 PPI active compounds with molecular weight < 1,200 and ≤ 10 μM for IC_50_, EC_50_, or *K*_d_ in the ChEMBL 23, and 52 “putative” inactive compounds from the vendor catalog. The second model (RF-TI) was created using 108 and 106 Keap1/Nrf2 PPI active and “true” inactive compounds respectively, based on activity values of AC_50_, IC_50_, EC_50_, *K*_d_, or Inhibition (%) from the DLiP database (https://skb-insilico.com/dlip) that is a curated collection of PPI activity data from public databases including ChEMBL, TIMBAL, and 2P2I. The training set for RF-PI and RF-TI composed of 1,277 and 1,518 features, respectively. The models were evaluated using fivefold cross-validation: all data were randomly split into five even test sets and the predicting performance of each test set by the model created from the data excluding the test set was evaluated. We used it in stratified manner (*i.e.*, the class balance of training and test sets of each fold was maintained as that of the entire set) with performance metrics: accuracy, precision, recall, specificity, and Matthews correlation coefficient (MCC):

1 $${\text{Accuracy}} = \frac{{{\text{TP}} + {\text{TN}}}}{{{\text{TP}} + {\text{TN}} + {\text{FP}} + {\text{FN}}}}$$

2 $${{\text{Precision}} = \frac{{{\text{TP}}}}{{{\text{TP}} + {\text{FP}}}}}$$

3 $${{\text{Recall}} = \frac{{{\text{TP}}}}{{{\text{TP}} + {\text{FN}}}}}$$

4 $${{\text{Specificity}} = \frac{{{\text{TN}}}}{{{\text{TN}} + {\text{FP}}}}}$$

5 $${{\text{MCC}} = \frac{{{\text{TP}} \times {\text{TN}} - {\text{FP}} \times {\text{FN}}}}{{\sqrt {\left( {{\text{TP}} + {\text{FP}}} \right)\left( {{\text{TP}} + {\text{FN}}} \right)\left( {{\text{TN}} + {\text{FP}}} \right)\left( {{\text{TN}} + {\text{FN}}} \right)} }}}$$

where TP, TN, FP, and FN denote the number of true positives, true negatives, false positives, and false negatives, respectively. The area under the receiver operating characteristic curve (AUCROC) and precision-recall curve (AUCPR) were used for evaluation of the performance without threshold. The value of MCC ranges from − 1 to 1, although those of the other metrics range from 0 to 1. Random predictions for balanced dataset show 0 for MCC and 0.5 for the other metrics, and good prediction models show higher values than those values.

### Bioassay materials

Library compounds were synthesized in the AMED project. The synthetic procedures of the hit compounds are described in Supplementary Method. The purity and MS information of the hit compounds were obtained via LC–MS on a Shimadzu LCMS-2020 [gradient from 5% MeCN/95% H_2_O to 100% MeCN/0% H_2_O (+ 0.05% trifluoroacetic acid) in 2 min with a Shim-pack XR-ODS column or gradient from 10% MeCN/90% H_2_O to 95% MeCN/5% H_2_O (+ 5 mM NH_4_HCO_3_) in 2 min with a Kinetex EVO C18 column] and were described in Supplementary Table S2. The purity of the samples was assessed using a UV detector at 254 nm. Nrf2 (TAMRA-LQLDEETGEFLPIQ-NH2) and F1325 (TAMRA-Abu(4)-VWYTDIRMRDWM) peptides were synthesized by TORAY Research Center. Streptavidin-Tb cryptate was purchased from Cisbio Bioassays.

### Preparation of human Keap1 and Bcl6 protein

The hKeap1 Kelch domain (residues Ala321–Thr609) and hBcl6 domain (residues Ala5–Glu129) were amplified by PCR using human cDNA libraries. Three cysteine residues of hBcl6 were then mutated (Cys8Gln, Cys67Arg, Cys84Asn) as reported^52^. Hereafter, this mutant is referred to as hBcl6. hKeap1 and hBcl6 were ligated into a pET21 vector (Merck Millipore) next to the His-Avi and His-Avi-SUMO-FLAG tags (LifeSensors), respectively. The proteins were expressed with isopropyl β-D-1-thiogalactopyranoside (IPTG) induction in *Escherichia coli* BL21 (DE3) (Nippon Gene). The proteins were purified using Ni–NTA (FUJIFILM Wako Pure Chemical) and Superdex 200 (GE Healthcare). Next, the purified proteins were enzymatically biotinylated in vitro. Briefly, the proteins were incubated for 3 h at 30 °C with purified *Escherichia coli* BirA in the presence of D-biotin, magnesium chloride (MgCl_2_), and ATP, which was replaced with final buffer (50 mM Tris-hydrochloride [HCl, pH 8.0], 150 mM sodium chloride [NaCl], and 5% glycerol). The proteins were concentrated and quantified using a TaKaRa bicinchoninic acid (BCA) protein assay kit (Takara Bio). The biotinylation rate of the Avi tag was calculated from its protein binding rate to streptavidin sepharose high-performance (GE Healthcare).

### PPI inhibition assay development using TR-FRET

Keap1/Nrf2 PPI inhibition and Bcl6/F1325 PPI inhibition counter assays were performed using TR-FRET as previously described^52,53^ to determine the potency of selected inhibitors using 384-well white flat-bottom small volume plates (Greiner Bio-One). Assay buffer consisting of 50 mM Tris–HCl (pH 7.5, FujiFilm Wako Pure Chemical), 100 mM NaCl (Nacalai Tesque), 0.01% Tween-20 (Bio-Rad), 1 mM dithiothreitol (FujiFilm Wako Pure Chemical), and 0.01% bovine serum albumin (Merck Millipore) was used to dilute the reagents. The test compounds were prepared in DMSO solution and dispensed into each well using the Echo 555 Liquid Handler (Labcyte). The biotinylated hKeap1 or hBcl6 proteins conjugated with streptavidin-Tb cryptate (final 0.5 nM) were added to each well, pre-incubated for 1 h, and then TAMRA-Nrf2 or TAMRA-F1325 peptides (final concentration, 6.0 and 12 nM, respectively) were added to each well, followed by incubation for 1 h at 24 °C in the dark. TR-FRET signals of individual wells were measured using an Envision multilabel plate reader (PerkinElmer; excitation wavelength [Ex] 337 nm, emission wavelength [Em] 570 nm/535 nm). To determine the inhibition rate, TR-FRET signal of the PPI between hKeap1 and Nrf2 peptides (counter assay: hBcl6 and F1325) was set as 0% inhibition and that of the Nrf2 peptide alone (counter assay: F1325 alone) was set as 100% inhibition. The test compound inhibition (%) was calculated as: [1 − (T_sample_ − T_100% inhibition_)/(T_0% inhibition_ − T_100% inhibition_)] × 100, where T indicates the TR-FRET signal.

### 3D alignment analysis

The 3D alignment model was constructed using the ligand-based method with Forge 10.6^54^. We used the X-ray crystal structure of the Nrf2 peptide in the human Keap1 Kelch domain (PDB ID: 2flu)^45^. The 3D conformations of the hit compounds confirmed by our assay were generated using the conformational hunt function of Forge. We used default settings, except for constraint on the negative charge of the positions, where contacts between Nrf2 and Keap1 were observed in the X-ray crystal structure. The sum of field and shape similarity scores was used as total score to rank predicted binding poses. Finally, representative binding poses were selected from the top five predicted poses by visual inspection as the common substructure forming the same conformation.

## Supplementary Information

Supplementary Information 1.

Supplementary Information 2.

Supplementary Information 3.

Supplementary Information 4.

Supplementary Information 5.

Supplementary Information 6.

Supplementary Information 7.

Supplementary Information 8.

Supplementary Information 9.

Supplementary Information 10.

## Data Availability

All data generated or analysed during this study are included in this published article (and its Supplementary Information files).
