# Supplementary material for: Identification of novel inhibitors of Keap1/Nrf2 by a promising method combining protein–protein interaction-oriented library and machine learning
Source: Sci Rep. 2021 Apr 1;11:7420. doi: 10.1038/s41598-021-86616-1 (PMC8016952; doi:10.1038/s41598-021-86616-1)
Supplement: Supplementary file 6 — Supplementary Information 6. [file 41598_2021_86616_MOESM6_ESM.pdf]

Supplementary Information

Identification of novel inhibitors of Keap1/Nrf2 by a promising method combining protein–protein interaction-oriented library and machine learning

Yugo Shimizu[1], Tomoki Yonezawa[1,2], Junichi Sakamoto[3], Toshio Furuya[4], Masanori Osawa[1], and Kazuyoshi Ikeda[1,*]

[1]Division of Physics for Life Functions, Keio University Faculty of Pharmacy, 1-5-30 Shibakoen, Minato-ku, Tokyo 105-8512, Japan

[2]Lifematics Inc., Sanshin-Hatchobori Bldg. 5F, 2-25-10, Hatchobori, Chuo-ku, Tokyo 104-0032, Japan

[3]Axcelead Drug Discovery Partners, Inc., 26-1, Muraoka-Higashi 2-chome, Fujisawa, Kanagawa 251-0012, Japan

[4]Drug Discovery Department, Research & Development Division, PharmaDesign, Inc., Hatchobori 2-19-8, Chuo-ku, Tokyo 104-0032, Japan.

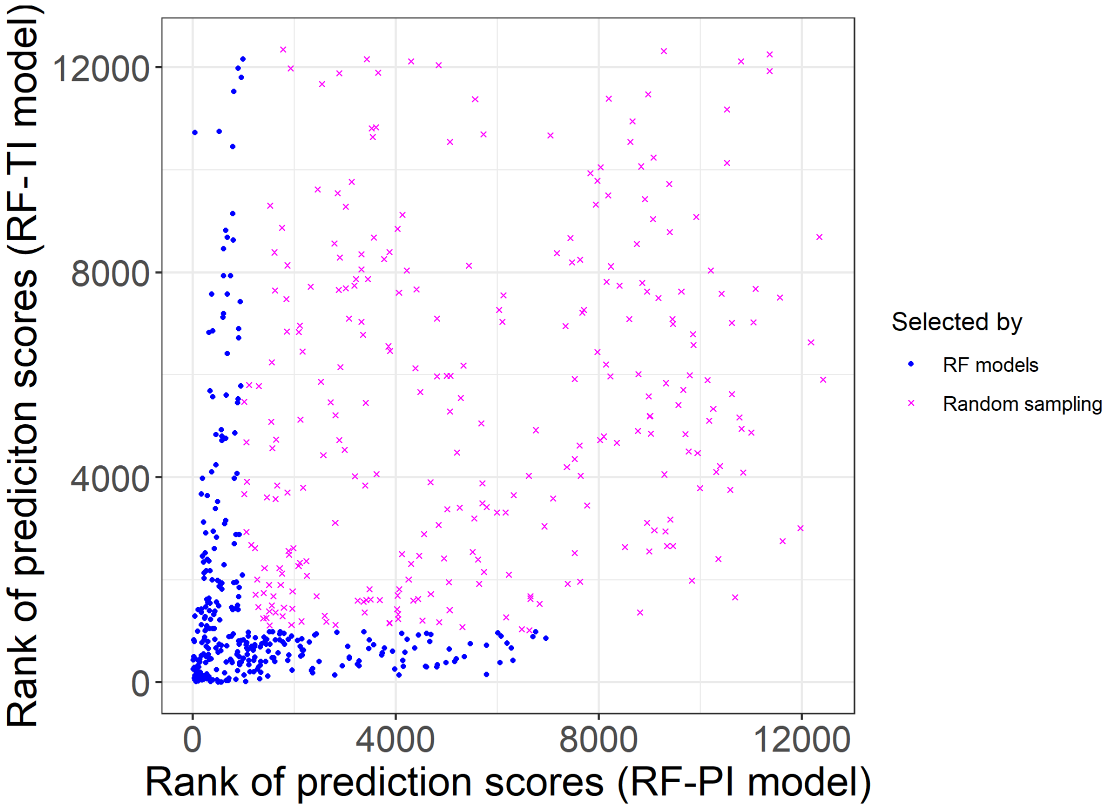

Figure S1. Distribution of ranks of prediction scores for RF-TI and RF-PI models of assayed compounds. Compounds selected by the RF models (N=329) are shown in blue and by random sampling (N=291) are shown in magenta.

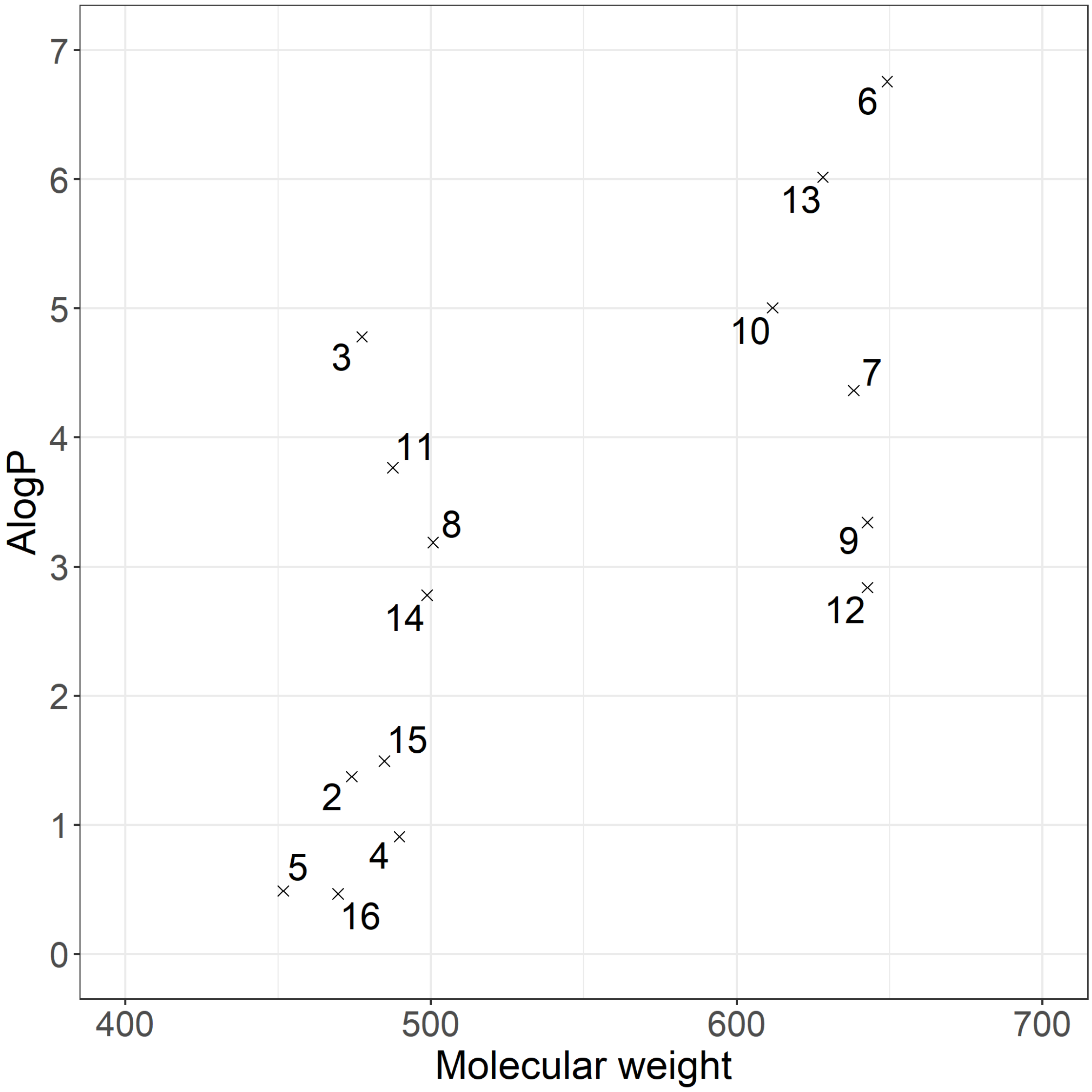

Figure S2. Distribution of molecular properties (molecular weight and AlogP) of 15 hit compounds (**2**–**16**).

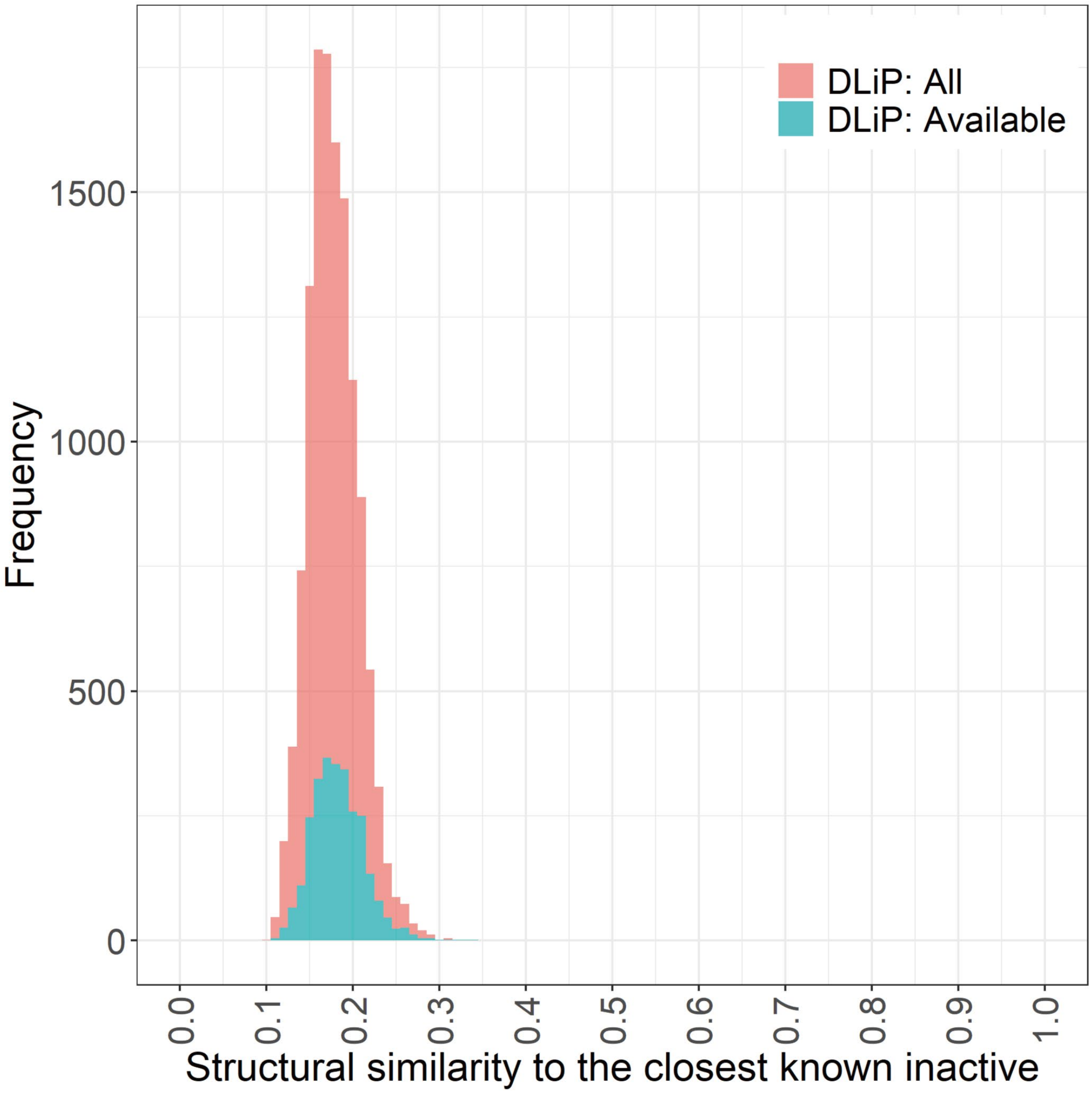

Figure S3. Distribution of structural similarities of all (N=12,593, light red) compounds of the DLiP library and their available subset in this study (N=2,684, cyan) to their closest known Keap1/Nrf2 PPI inactives in the databases.

Supplementary Method

Synthetic procedures of the hit compounds

The tested compounds were synthesized by combinatorial chemistry. The building blocks (BBs) of the hit compounds (**1**–**16**) are shown in Table S1. Their synthetic procedures for connecting BBs to obtain the compounds (**1**–**16**) are classified into five reaction types: two compositions, three compositions, Amine, COOH, and COOH + Amine (Table S1). The identity of the compounds was confirmed, based on the MS data of the hit compounds (Table S2) and the MS/NMR data of the BBs used for their synthesis (Tables S3). The synthetic procedures of the five reaction types are shown below.

The synthetic procedures of reaction type: Two compositions (Compound **16**)

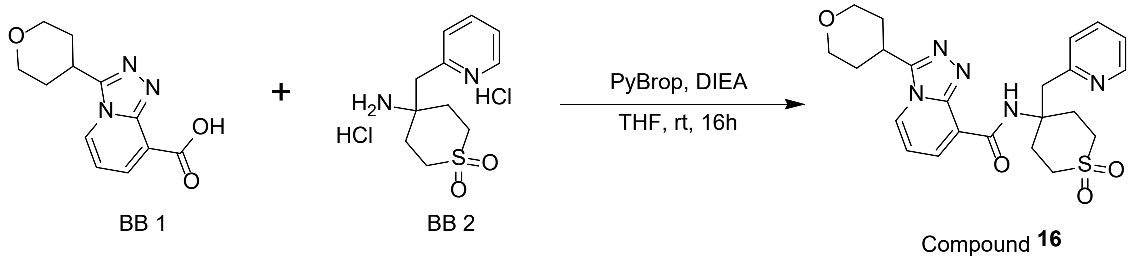

Scheme S1.

To a stirred solution of 3-(oxan-4-yl)-[1,2,4]triazolo[4,3-a]pyridine-8-carboxylic acid (25 mg, 0.10 mM, 1.00 equiv) and 4-amino-4-(pyridin-2-ylmethyl)-1lambda6-thiane-1,1-dione dihydrochloride (32 mg, 0.10 mM, 1.01 equiv) in THF (2 mL) was added PyBrop (70mg, 0.15 mM, 1.5 equiv) and DIEA (38 mg, 0.29 mM, 2.91 equiv) at rt. The reaction was stirred at rt for 16 hours. The resulting mixture was concentrated under reduced pressure. The residue was purified by Prep-HPLC with the following conditions: Column, XBridge Shield RP18 OBD Column, 30*150 mm, 5 µm; mobile phase, Water (10 mM/L NH_4HCO_3 + 0.1% $NH_3.H_2O$) and CH_3CN (15% Phase B up to 45% in 8 min); Detector, UV, 254 nm, to afford N-[1,1-dioxo-4-(pyridin-2-ylmethyl)-1lambda6-thian-4-yl]-3-(oxan-4-yl)-[1,2,4]triazolo[4,3-a]pyridine-8-carboxamide (6.8 mg, 14.32%) as a colorless semi-solid.

The synthetic procedures of reaction type: Three compositions (Compound **6**)

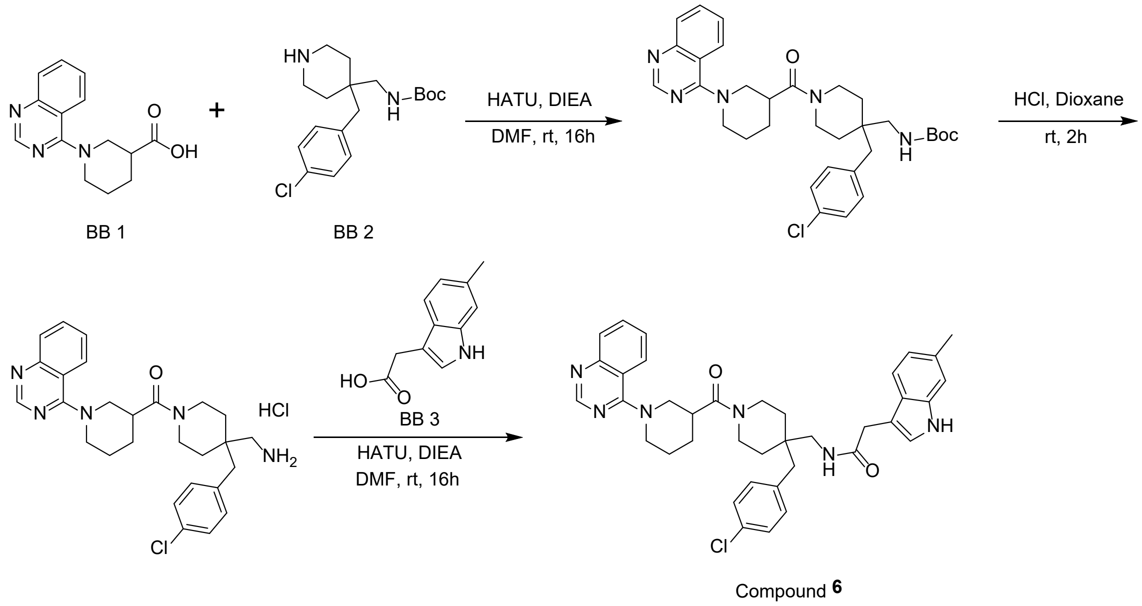

Scheme S2.

To a stirred solution of 1-(quinazolin-4-yl)piperidine-3-carboxylic acid (25.72 mg, 0.100 mM, 1.00 equiv) and tert-butyl N-({4-[(4-chlorophenyl)methyl]piperidin-4-yl}methyl)carbamate (33.9 mg, 0.100 mM, 1.00 equiv) in DMF (3 mL) was added HATU (57.01 mg, 0.150 mM, 1.5 equiv) and DIEA (38.76 mg, 0.300 mM, 3 equiv). The resulting mixture was stirred at rt for 16 hours. The resulting mixture was concentrated under vacuum. The residue was applied onto a silica gel column with dichloromethane/methanol (10:1~5:1). This resulted in tert-butyl N- ({4-[(4-chlorophenyl)methyl]-1-[1-(quinazolin-4-yl)piperidine-3-carbonyl]piperidin-4-yl}methyl)carbamate (28.9 mg, 49.92%). To a stirred solution of tert-butyl N-({4-[(4-chlorophenyl)methyl]-1-[1-(quinazolin-4-yl)piperidine-3-carbonyl]piperidin-4-yl}methyl)carbamate (28.9 mg, 0.05 mM, 1.00 equiv) in 1,4-dioxane (1 mL) was added HCl (4M in 1,4-dioxane) (1 mL) at rt. The resulting mixture was stirred at rt for 2 hours. The resulting mixture was concentrated under vacuum. This resulted in 1-{4-[(4-chlorophenyl)methyl]-1-[1-(quinazolin-4-yl)piperidine-3-carbonyl]piperidin-4-yl}methanamine hydrochloride (26 mg). To a stirred solution of 1-{4-[(4-chlorophenyl)methyl]-1-[1-(quinazolin-4-yl)piperidine-3-carbonyl]piperidin-4-yl}methanamine hydrochloride (26 mg, 0.05 mM, 1.00 equiv) and (6-methyl-1H-indol-3-yl)acetic acid (9.44 mg, 0.05 mM, 1.0 equiv) in DMF (1 mL) was added HATU (28.44 mg, 0.075 mM, 1.5 equiv) and DIEA (19.33 mg, 0.15 mM, 3 equiv) at rt. The resulting mixture was stirred at rt for 16 hours. The resulting mixture was concentrated under vacuum. The crude product was purified by Prep-HPLC with the following conditions:

Column, XBridge Shield RP18 OBD Column, 30*150 mm, 5 µm; mobile phase, Water (10 mM/L NH_4HCO_3 + 0.1%$NH_3.H_2O$) and CH_3CN (15% Phase B up to 45% in 8 min); Detector, UV, 254 nm, to afford N-({4-[(4-chlorophenyl)methyl]-1-[1-(quinazolin-4-yl)piperidine-3-carbonyl]piperidin-4-yl}methyl)-2-(6-methyl-1H-indol-3-yl)acetamide (5.6 mg, 17.3%) as a purple solid.

The synthetic procedures of reaction type: Amine (Compound **4**)

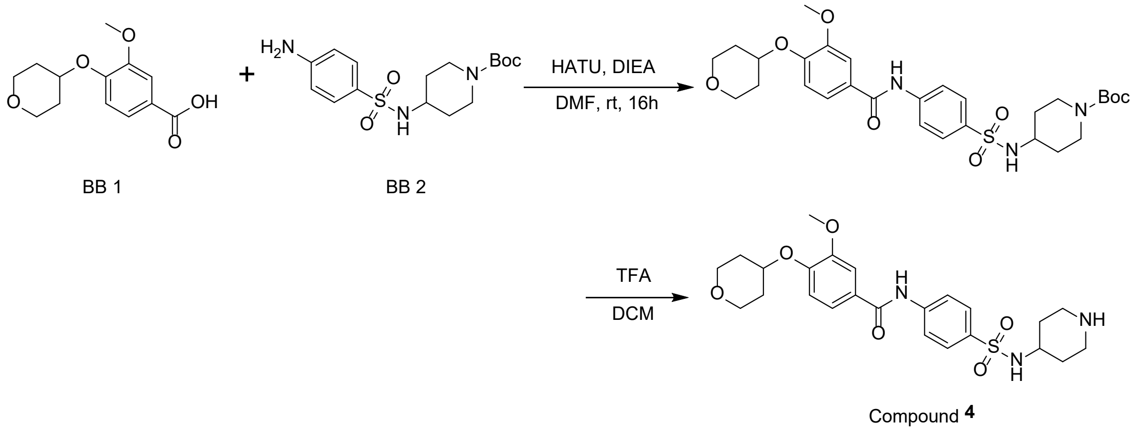

Scheme S3.

To a stirred solution of 3-methoxy-4-(oxan-4-yloxy)benzoic acid (25.3 mg, 0.100 mM, 1.00 equiv) and tert-butyl 4-(4-aminobenzenesulfonamido)piperidine-1-carboxylate (35.65 mg, 0.100 mM, 1 equiv) in *N*,*N*-dimethylformamide (DMF) (2 mL) was added *N*,*N*-diisopropylethylamine (DIEA) (38.89 mg, 0.300 mM, 3 equiv) and 1-[Bis(dimethylamino)methylene]-1*H*-1,2,3-triazolo[4,5-*b*]pyridinium 3-Oxide Hexafluorophosphate (HATU) (57.20 mg, 0.150 mM, 1.5 equiv). The resulting mixture was stirred at rt for 16 hours. The resulting mixture was concentrated under reduced pressure. The residue was applied onto a silica gel column with dichloromethane/methanol (10:1~5:1). This resulted in tert-butyl 4-{4-[3-methoxy-4-(oxan-4-yloxy)benzamido]benzenesulfonamido}piperidine-1-carboxylate (23 mg, 38.89%). To a stirred solution of tert-butyl 4-{4-[3-methoxy-4-(oxan-4-yloxy)benzamido]benzenesulfonamido}piperidine-1-carboxylate (23 mg, 0.039 mM, 1.00 equiv) in dichloromethane (1 mL) was added TFA (1 mL). The resulting mixture was stirred at rt for 2 hours. The resulting mixture was concentrated under reduced pressure. The residue was purified by Prep-HPLC with the following conditions: Column, XBridge Shield RP18 OBD Column, 30*150 mm, 5 µm; mobile phase, Water (10 mM/L NH_4HCO_3 + 0.1% $NH_3.H_2O$) and CH_3CN (15% Phase B up to 45% in 8 min); Detector, UV, 254 nm, to afford 3-methoxy-4-(oxan-4-yloxy)-N-{4-[(piperidin-4-yl)sulfamoyl]phenyl}benzamide (4.2 mg, 21.99%) as a white solid.

The synthetic procedures of reaction type: COOH (Compound **3**)

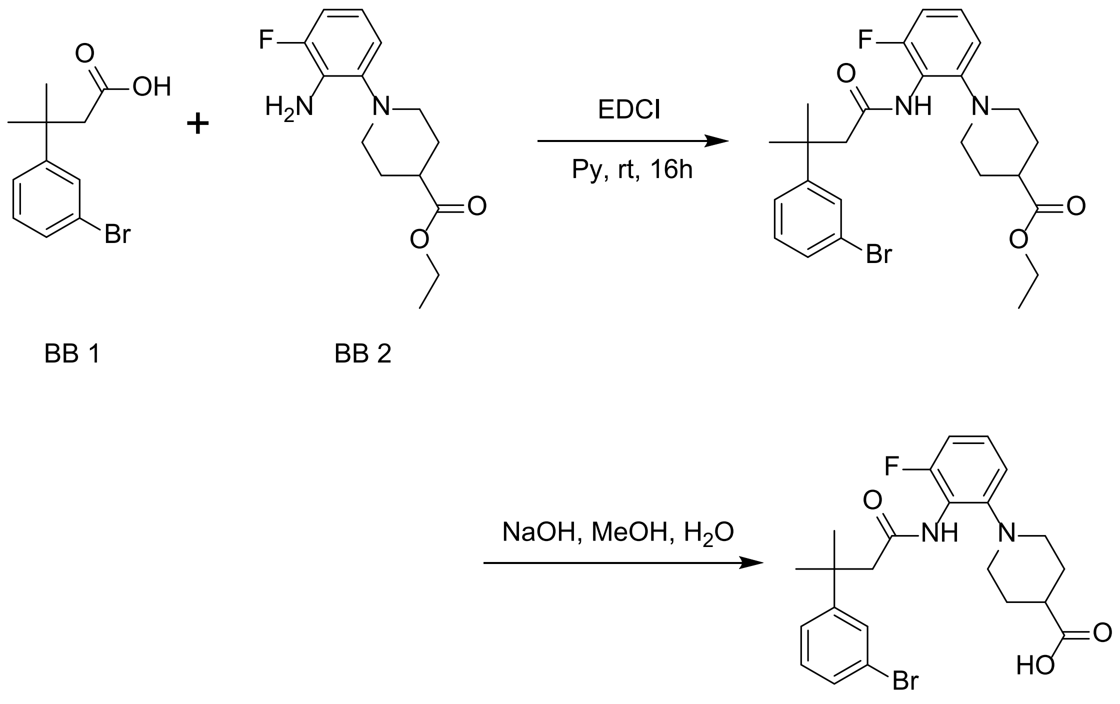

Compound **3**

Scheme S4.

To a stirred mixture of 3-methyl-3-phenylbutanoic acid (17.82 mg, 0.100 mM, 1.00 equiv) and ethyl 1-(2-amino-3-fluorophenyl)piperidine-4-carboxylate (26.63 mg, 0.100 mM, 1 equiv) in pyridine (2 mL) was added 1-ethyl-3-(3-dimethylaminopropyl)carbodiimide (EDCI), (38.34 mg, 0.2 mM, 2 equiv). The resulting mixture was stirred at rt for 16 hours. The resulting mixture was concentrated under vacuum. This resulted in 100 mg crude ethyl 1-(2-(3-(3-bromophenyl)-3-methylbutanamido)-3-fluorophenyl)piperidine-4-carboxylate. To a stirred mixture of ethyl 1-(2-(3-(3-bromophenyl)-3-methylbutanamido)-3-fluorophenyl)piperidine-4-carboxylate (100 mg crude) in CH_3OH (1 mL) was added NaOH (10 mg, 0.5 mM, 2.5 equiv) in 1 mL H_2O at room temperature. The resulting mixture was stirred overnight at room temperature. The resulting mixture was concentrated under vacuum. The residue was purified by Prep-HPLC with the following conditions: Column, Xselect CSH OBD Column 30*150 mm 5 µm, n; mobile phase, Water (0.1% FA) and CH_3CN (30% Phase B up to 60% in 10 min), Detector, UV, 254 nm, to afford 1-{2-[3-(3-bromophenyl)-3-methylbutanamido]-3-fluorophenyl}piperidine-4-carboxylic acid (1.5 mg, 3.14%) as a white solid.

The synthetic procedures of reaction type: COOH + Amine (Compound **1**)

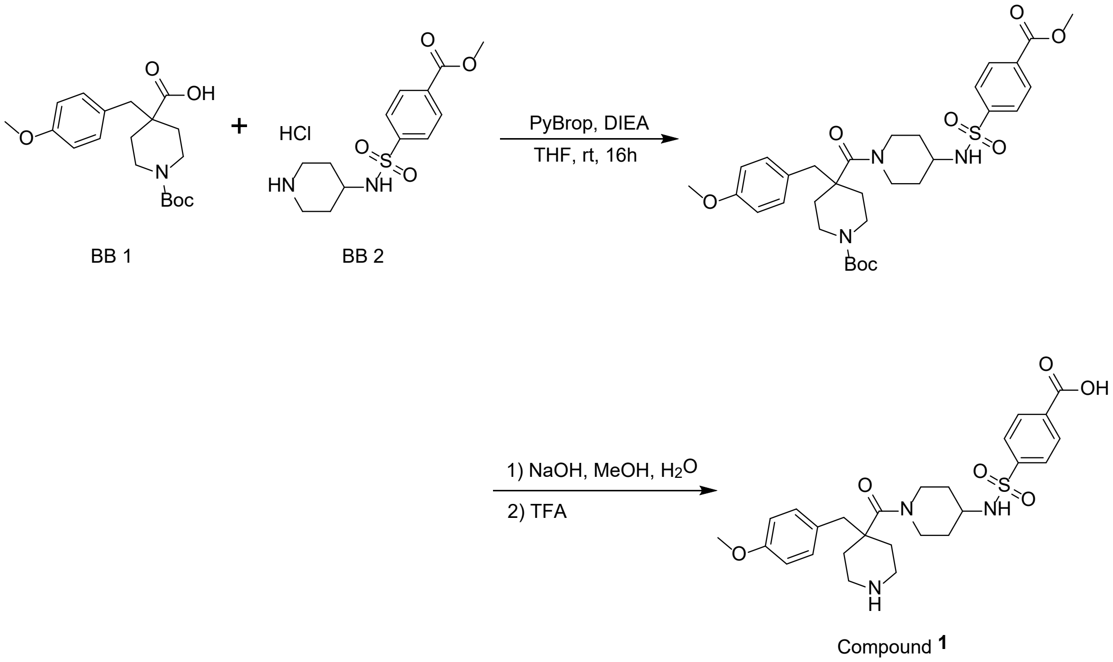

Scheme S5.

To a stirred mixture of 1-(tert-butoxycarbonyl)-4-[(4-methoxyphenyl)methyl]piperidine-4-carboxylic acid (34.94 mg, 0.100 mM, 1.00 equiv) and methyl 4-[(piperidin-4-yl)sulfamoyl]benzoate hydrochloride (33.48 mg, 0.100 mM, 1.0 equiv) in tetrahydrofuran (THF) (2 mL) was added PyBrop (70 mg, 0.15 mM, 1.5 equiv) and *N*,*N*-diisopropylethylamine (DIEA) (38 mg, 0.29 mM, 2.91 equiv) at rt. The reaction was stirred at rt for 16 hours. The resulting mixture was concentrated under vacuum. This resulted in 150 mg crude tert-butyl 4-{4-[4-(methoxycarbonyl)benzenesulfonamido]piperidine-1-carbonyl}-4-[(4-methoxyphenyl)methyl]piperidine-1-carboxylate. To a stirred mixture of tert-butyl 4-{4-[4-(methoxycarbonyl)benzenesulfonamido]piperidine-1-carbonyl}-4-[(4-methoxyphenyl)methyl]piperidine-1-carboxylate (150 mg crude) in CH_3OH (1 mL) was added NaOH (10 mg, 0.25 mM, 2.5 equiv) in 1 mL H_2O at room temperature. The resulting mixture was stirred overnight at room temperature. The resulting mixture was concentrated under vacuum and added trifluoroacetic acid (TFA) (1 mL) at room temperature. The reaction was stirred at rt for 2 hours. The resulting mixture was concentrated under vacuum. The residue was purified by Prep-HPLC with the following conditions: Column, Xselect CSH OBD Column 30*150 mm 5 μm, n; mobile phase, Water (0.1% FA) and ACN (30% Phase B up to 60% in 10 min), Detector, UV, 254 nm, to afford 4-[(1-{4-[(4-methoxyphenyl)methyl]piperidine-4-carbonyl}piperidin-4-yl)sulfamoyl]benzoic acid (1.6 mg, 3.15%) as a light brown solid.

Compounds	Reaction type	BB1	BB2	BB3
**1**	COOH + Amine	KSD-K-014-148-0	KSD-K-002-152-0	
**2**	COOH + Amine	KSD-K-008-340-0	KSD-K-014-139-0	
**3**	COOH	KSD-K-050-3503-0	KSD-K-008-340-0	
**4**	Amine	KSD-K-013-1208-0	KSD-K-002-161-0	
**5**	COOH + Amine	KSD-K-014-147-0	KSD-K-008-310-0	
**6**	Three compositions	KSD-K-026-082-0	KSD-K-032-385-0	KSD-K-050-4159-0
**7**	Three compositions	KSD-K-026-082-0	KSD-K-032-385-0	KSD-K-050-3264-0
**8**	COOH	KSD-K-008-310-0	KSD-K-050-3285-0	
**9**	Three compositions	KSD-K-002-430-0	KSD-K-017-1579-0	KSD-K-013-2652-0
**10**	Three compositions	KSD-K-026-082-0	KSD-K-032-382-0	KSD-K-013-1730-0
**11**	COOH	KSD-K-050-119-0	KSD-K-008-310-0	
**12**	Three compositions	KSD-K-002-430-0	KSD-K-017-1579-0	KSD-K-013-2674-0
**13**	Three compositions	KSD-K-050-117-0	KSD-K-032-392-0	KSD-K-050-4159-0
**14**	COOH	KSD-K-050-1469-0	KSD-K-002-149-0	
**15**	Amine	KSD-K-014-202-0	KSD-K-050-1290-0	
**16**	Two compositions	KSD-K-013-1935-0	KSD-K-050-3326-0	

Table S1. The reaction types and BBs used in the synthesis of the hit compounds (**1**–**16**)

Compounds	Molecular weight	MS (m/z)	Purity (%)	HPLC methods[a]
**1**	515.62	516.15	98.9	TFA
**2**	473.97	474.25	99.6	NH_4HCO_3
**3**	477.37	479.05	95.9	TFA
**4**	489.58	490.15	98.7	NH_4HCO_3
**5**	451.56	452.20	99.8	TFA
**6**	649.22	649.20	91.8	TFA
**7**	638.22	638.10	99.6	NH_4HCO_3
**8**	500.61	501.10	98.6	TFA
**9**	642.72	643.20	97.4	TFA
**10**	611.71	612.25	98.6	TFA
**11**	487.39	489.05	99.8	TFA
**12**	642.72	643.20	97.8	TFA
**13**	628.20	628.15	99.0	NH_4HCO_3
**14**	498.58	499.15	95.6	TFA
**15**	484.61	485.10	99.7	TFA
**16**	469.56	470.15	98.5	TFA

Table S2. MS data and HPLC purity of the hit compounds. [a]TFA: gradient from 5% CH_3CN/95% H_2O to 100% CH_3CN/0% H_2O (+0.05% trifluoroacetic acid) in 2 min. NH_4HCO_3: gradient from 10% CH_3CN/90% H_2O to 95% CH_3CN/5% H_2O (+5mM NH_4HCO_3) in 2 min.

BB	Structure	mp (°C)	Molecular weight	MS (*m*/*z*)
KSD-K-014-148-0	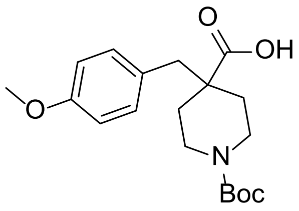	218–220	349.42	[M+1-Boc]: 250
KSD-K-002-152-0	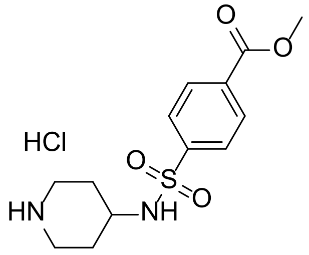	266–268	334.82	[M+1-HCl]: 299
KSD-K-008-340-0	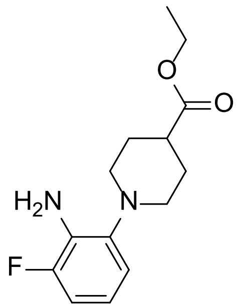	38–41	266.32	[M+1]: 267
KSD-K-014-139-0	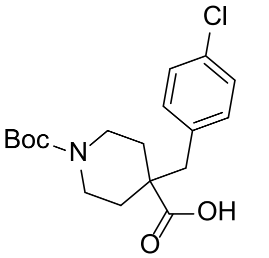	185–187	353.84	[M-1]: 352
KSD-K-050-3503-0	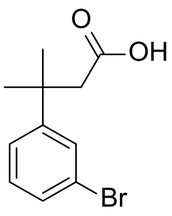	92–94	257.12	[M-1]: 255 and 257
KSD-K-013-1208-0	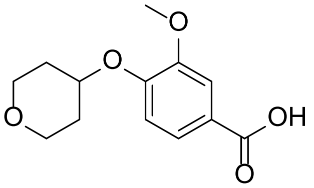	138–140	252.26	[M-1]: 251

KSD-K-002-161-0	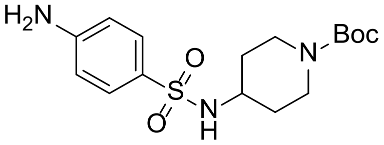	76–78	355.45	[M+Na]: 378
KSD-K-014-147-0	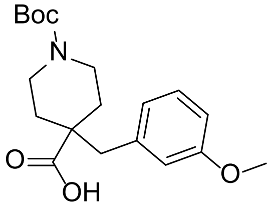	205–207	349.42	[M+1-Boc]: 250
KSD-K-008-310-0	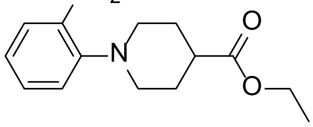	46–49	248.33	[M+1]: 249
KSD-K-026-082-0	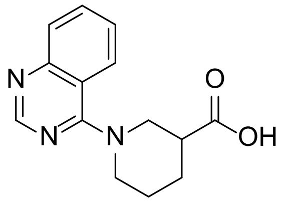	160–162	257.29	[M+1]: 258
KSD-K-032-385-0	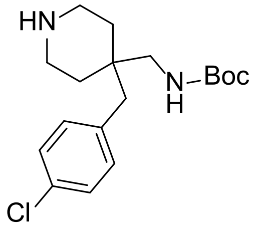	97–101	338.88	[M+1]: 339
KSD-K-050-4159-0	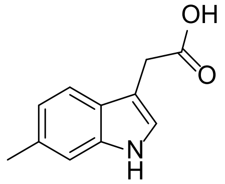	174–176	189.21	[M+1]: 190
KSD-K-050-3264-0	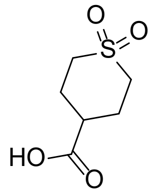	176–178	178.21	[M-1]: 177

KSD-K-050-3285-0	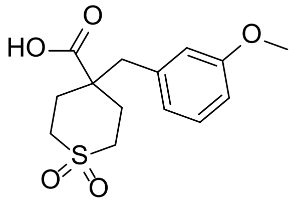	70–74	298.35	[M-1]: 297
KSD-K-002-430-0	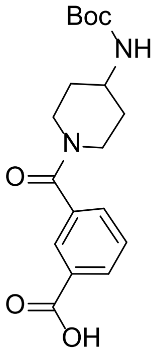	155–160	348.39	[M+1-t-Bu]: 293
KSD-K-017-1579-0	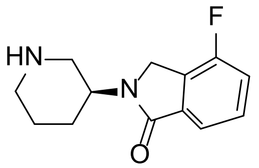	103–105	234.27	[M+1]: 235
KSD-K-013-2652-0	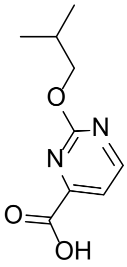	148–150	196.2	[M+1]: 197
KSD-K-032-382-0	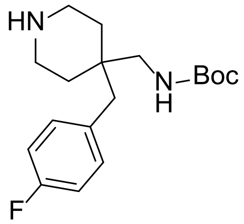	170–172	322.42	[M+1]: 323
KSD-K-013-1730-0	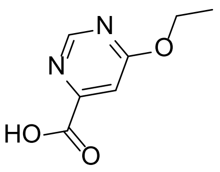	180–182	168.15	[M+1]: 169
KSD-K-050-119-0	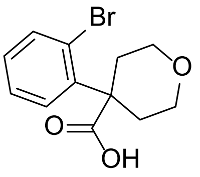	242–244	285.13	[M-1]: 283 and 285

KSD-K-013-2674-0	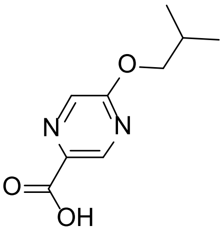	133–135	196.2	[M+1]: 197
KSD-K-050-117-0	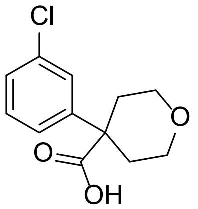	138–140	240.68	[M-1]: 239
KSD-K-032-392-0	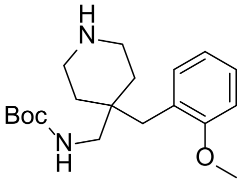	54–56	334.45	[M+1]: 335
KSD-K-050-1469-0	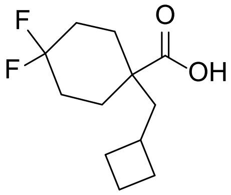	44–46	232.27	212, 204, 187
KSD-K-002-149-0	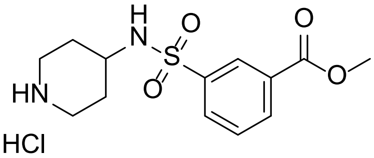	250–253	334.82	[M+1-HCl]: 299
KSD-K-014-202-0	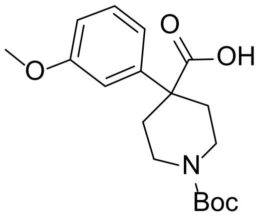	112–114	335.39	[M+1-Boc]: 236
KSD-K-050-1290-0	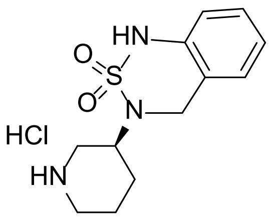	252–254	303.81	[M+1-HCl]: 268

KSD-K-013-1935-0	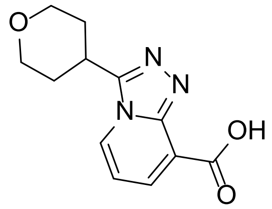	193–196	247.25	[M+1]: 248
KSD-K-050-3326-0	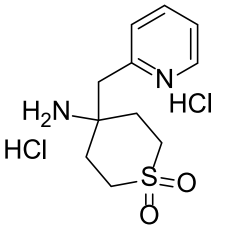	264–266	313.24	[M+1-2HCl]: 241

Table S3. The structures, mp, and MS data of BBs used in the synthesis.

NMR data of BBs

KSD-K-014-148-0:
^{1}H NMR (300 MHz, CD_3OD): δ 6.96–6.93 (d, 8.7 Hz, 2H), 6.73–6.69 (m, 2H), 3.83–3.82 (d, 3.6 Hz, 2H), 3.65 (s, 3H), 2.78–2.69 (m, 4H), 1.93–1.88 (d, 13.5 Hz, 2H), 1.34 (s, 9H), 1.30–1.25 (m, 2H).

KSD-K-002-152-0:
^{1}H NMR (300 MHz, D_2O): δ 8.12–8.09 (d, 8.4 Hz, 2H), 7.93–7.90 (d, 8.4 Hz, 2H), 3.89 (s, 3H), 3.45–3.38 (m, 1H), 3.36–3.25 (m, 2H), 2.98–2.88 (m, 2H), 1.90–1.85 (m, 2H), 1.68–1.55 (m, 2H).

KSD-K-008-340-0:
^{1}H NMR (300 MHz, DMSO): δ 6.90–6.69 (m, 2H), 6.55 (td, J = 8.1, 6.2 Hz, 1H), 4.61 (s, 2H), 4.11 (q, J = 7.1 Hz, 2H), 3.07 (s, 2H), 2.59 (t, J = 10.5 Hz, 2H), 2.44 (d, J = 11.0 Hz, 1H), 2.04–1.66 (m, 4H), 1.21 (t, J = 7.1 Hz, 3H).

KSD-K-014-139-0:
^{1}H NMR (400 MHz, DMSO): δ 12.65 (s, 1H), 7.34–7.31 (d, 8.4 Hz, 2H), 7.14–7.12 (d, 8.4 Hz, 2H), 3.79–3.76 (m, 2H), 2.90–2.70 (m, 4H), 1.86–1.82 (m, 2H), 1.38–1.31 (m, 11H).

KSD-K-050-3503-0:
^{1}H NMR (300 MHz, $CDCl_3$): δ 7.49–7.48 (m, 1H), 7.35–7.27 (m, 2H), 7.21–7.15 (m, 1H), 2.64 (s, 2H), 1.44 (s, 6H).

KSD-K-013-1208-0:
^{1}H NMR (300 MHz, $CDCl_3$): δ 7.53–7.50 (d, 8.4 Hz, 1H), 7.45 (s, 1H), 7.15–7.12 (d, 8.4 Hz, 1H), 4.63 (m, 1H), 3.87–3.80 (m, 5H), 3.50–3.43 (m, 2H), 1.99–1.94 (m, 2H), 1.61–1.57 (m, 2H).

KSD-K-002-161-0:
^{1}H NMR (300 MHz, $CDCl_3$): δ 7.69–7.64 (m, 2H), 6.72–6.67 (m, 2H), 4.52–4.49 (d, 7.5 Hz, 1H), 4.17 (s, 2H), 3.92–3.87 (m, 2H), 3.27–3.23 (m, 1H), 2.85–2.77 (m, 2H), 1.78–1.73 (m, 2H), 1.44 (s, 9H), 1.39–1.22 (m, 2H).

KSD-K-014-147-0:
^{1}H NMR (400 MHz, DMSO): δ 12.60 (s, 1H), 7.19–7.15 (m, 1H), 6.79–6.77 (d, 8.4 Hz, 1H), 6.70–6.68 (m, 2H), 3.78–3.72 (m, 5H), 2.80–2.76 (m, 4H), 1.87–1.83 (d, 13.6 Hz, 2H), 1.41–1.31 (m, 11H).

KSD-K-008-310-0:
^{1}H NMR (300 MHz, DMSO): δ 6.87 (d, J = 7.8 Hz, 1H), 6.79 (t, J = 7.6 Hz, 1H), 6.67 (dd, J = 8.0, 1.8 Hz, 1H), 6.53 (t, J = 7.5 Hz, 1H), 4.71 (s, 2H), 4.21–3.99 (m, 2H), 3.02 (dd, J = 9.5, 5.4 Hz, 2H), 2.57 (d, J = 11.2 Hz, 2H), 2.43 (dq, J = 10.5, 5.2, 3.9 Hz, 1H), 1.92 (d, J = 13.3 Hz, 2H), 1.86–1.64 (m, 2H), 1.21 (t, J = 7.1 Hz, 3H).

KSD-K-026-082-0:
^{1}H NMR (300 MHz, DMSO): δ 12.41 (s, 1H), 8.59 (s, 1H), 7.98–7.95 (d, J = 8.4 Hz, 1H),7.78–7.74 (m, 2H), 7.54–7.48 (m, 1H), 4.25–4.19 (d, J = 16.2 Hz, 1H), 4.01–3.96 (d, J = 12.9 Hz, 1H), 3.50–3.30 (m, 2H), 2.72–2.64 (m, 1H), 2.02–1.93 (m, 1H), 1.80–1.58 (m, 3H)

KSD-K-032-385-0:
^{1}H NMR (300 MHz, DMSO): δ 7.40–7.26 (m, 2H), 7.18 (d, J = 8.2 Hz, 2H), 6.88 (t, J = 6.2 Hz, 1H), 2.87 (d, J = 6.1 Hz, 2H), 2.73 (t, J = 5.6 Hz, 4H), 2.56 (s, 2H), 1.39 (s, 9H), 1.19 (ddt, J = 25.4, 13.1, 6.8 Hz, 4H).

KSD-K-050-4159-0:
^{1}H NMR (300 MHz, DMSO): δ 12.11 (s, 1H), 10.73 (s, 1H), 7.37–7.35 (d, J = 7.8 Hz, 1H),7.12 (s, 2H), 6.82–6.79 (d, J = 7.8 Hz, 1H), 3.59 (s, 2H), 2.37 (s, 3H).

KSD-K-050-3264-0:
^{1}H NMR (300 MHz, CD_3OD): δ 3.19–3.05 (m, 4H), 2.76–2.66 (m, 1H), 2.50–2.20 (m, 4H).

KSD-K-050-3285-0:
^{1}H NMR (400 MHz, DMSO): δ 13.05 (s, 1H), 7.20 (t, J = 8.1 Hz, 1H), 6.81 (ddd, J = 8.3, 2.5, 1.0 Hz, 1H), 6.77–6.70 (m, 2H), 3.73 (s, 3H), 3.15 (d, J = 13.9 Hz, 2H), 3.00–2.88 (m, 2H), 2.86 (s, 2H), 2.28–2.20 (m, 2H), 1.94 (ddd, J = 14.9, 12.1, 3.1 Hz, 2H).

KSD-K-002-430-0:
^{1}H NMR (300 MHz, DMSO): δ 13.13 (s, 1H), 8.03–7.95 (m, 1H), 7.86 (s, 1H), 7.62–7.54 (m, 2H), 6.89–6.86 (d, 7.5 Hz, 1H), 4.40–4.20 (m, 1H), 3.60–3.41 (m, 2H), 3.20–2.91 (m, 2H), 1.91–1.61 (m, 2H), 1.44–1.18 (m, 11H).

KSD-K-017-1579-0:
^{1}H NMR (300 MHz, CD_3OD): δ 7.60–7.51 (m, 2H), 7.37–7.34 (m, 1H), 4.61–4.59 (d, 3 Hz, 2H), 4.27–4.19 (m, 1H), 3.13–2.98 (m, 2H), 2.83–2.75 (m, 1H), 2.61–2.51 (m, 1H), 2.03–1.91 (m, 1H), 1.90–1.80 (m, 2H), 1.80–1.65 (m, 1H).

KSD-K-013-2652-0:
^{1}H NMR (400 MHz, DMSO): δ 8.83–8.82 (d, 4.8 Hz, 1H), 7.59–7.58 (d, 4.8 Hz, 1H), 4.14–4.13 (d, 6.4 Hz, 2H), 2.10–2.03 (m, 1H), 1.00–0.98 (d, 6.8 Hz, 6H).

KSD-K-032-382-0:
^{1}H NMR (400 MHz, CD_3OD): δ 7.32–7.24 (m, 2H), 7.17–7.07 (m, 2H), 3.15–3.05 (m, 5H), 2.75 (s, 2H), 1.63–1.47 (m, 13H).

KSD-K-013-1730-0:
^{1}H NMR (300 MHz, DMSO): δ 8.85 (s, 1H), 7.28 (s, 1H), 4.45–4.36 (m, 2H), 1.36–1.28 (m, 3H).

KSD-K-050-119-0:
^{1}H NMR (300 MHz, CD_3OD): δ 7.62–7.54 (m, 2H), 7.43–7.37 (m, 1H), 7.21–7.15 (m, 1H), 4.01–3.92 (m, 2H), 3.81–3.73 (m, 2H), 2.55–2.47 (m, 2H), 2.25–2.15 (m, 2H).

KSD-K-013-2674-0:
^{1}H NMR (300 MHz, DMSO): δ 13.33 (s, 1H), 8.78 (s, 1H), 8.38 (s, 1H), 4.18–4.16 (d, 6.9 Hz, 2H), 2.13–2.01 (m, 1H), 1.00–0.98 (d, 6.6 Hz, 6H).

KSD-K-050-117-0:
^{1}H NMR (300 MHz, CD_3OD): δ 7.55–7.15 (m, 4H), 4.00–3.80 (m, 2H), 3.65–3.55 (m, 2H), 2.55–2.40 (m, 2H), 2.00–1.82 (m, 2H).

KSD-K-032-392-0:
^{1}H NMR (300 MHz, CD_3OD): δ 7.29–7.19 (m, 1H), 7.11 (dd, J = 7.5, 1.8 Hz, 1H), 6.98 (d, J = 8.2 Hz, 1H), 6.96–6.84 (m, 1H), 3.84 (s, 3H), 3.01 (d, J = 7.3 Hz, 6H), 2.69 (s, 2H), 1.46 (s, 13H).

KSD-K-050-1469-0:
^{1}H NMR (300 MHz, DMSO): δ 12.46 (s, 1H), 2.37–2.21 (m, 1H), 2.03–1.85 (m, 6H), 1.82–1.58 (m, 8H), 1.49–1.47 (m, 2H).

KSD-K-002-149-0:
^{1}H NMR (300 MHz, D_2O): δ 8.35 (s, 1H), 8.20–8.16 (d, 8.1 Hz, 1H), 8.05–8.02 (d, 8.1 Hz, 1H), 7.68–7.63 (m, 1H), 3.87 (s, 3H), 3.39–3.31 (m, 1H), 3.29–3.22 (m, 2H), 2.95–2.86 (m, 2H), 1.87–1.81 (m, 2H), 1.65–1.52 (m, 2H).

KSD-K-014-202-0:
^{1}H NMR (400 MHz, $CDCl_3$): δ 7.33–7.29 (m, 1H), 7.03–6.98 (m, 2H), 6.87–6.84 (m, 1H), 3.98–3.94 (m, 2H), 3.83 (s, 3H), 3.15–3.09 (m, 2H), 2.53–2.49 (m, 2H), 1.94–1.86 (m, 2H), 1.47 (s, 9H).

KSD-K-050-1290-0:
^{1}H NMR (300 MHz, DMSO): δ 10.48 (s, 1H), 9.24–9.11 (m, 1H), 8.96–8.80 (m, 1H), 7.23–7.13 (m, 2H), 7.03–6.98 (m, 1H), 6.86–6.70 (m, 1H), 4.72 (s, 2H), 3.97–3.90 (m, 1H), 3.16–3.08 (m, 2H), 3.02–2.90 (m, 1H), 2.72–2.66 (m, 1H), 2.00–1.60 (m, 4H).

KSD-K-013-1935-0:

^{1}H NMR (300 MHz, CD_3OD): δ 8.99 (dd, J = 6.9, 1.0 Hz, 1H), 8.54 (dd, J = 7.1, 1.0 Hz, 1H), 7.52 (t, J = 7.0 Hz, 1H), 4.11 (dt, J = 11.6, 3.5 Hz, 2H), 3.72 (ddd, J = 13.6, 10.5, 6.1 Hz, 3H), 2.09 (td, J = 10.2, 8.9, 3.7 Hz, 4H).

KSD-K-050-3326-0:

^{1}H NMR (300 MHz, DMSO): δ 8.66 (s, 4H), 8.03 (s, 1H), 7.67 (s, 1H), 7.54 (s, 1H), 3.60–3.30 (m, 6H), 2.38–1.97 (m, 4H).
